# Association Between Systemic Lupus Erythematosus and Myasthenia Gravis: Coincidence or Sequelae?

**DOI:** 10.7759/cureus.8422

**Published:** 2020-06-03

**Authors:** Sumit Raut, Ishani Reddy, Faryal Mustansir Sahi, Ayesha Masood, Bilal Haider Malik

**Affiliations:** 1 Internal Medicine, California Institute of Behavioral Neurosciences and Psychology, Fairfield, USA; 2 Psychiatry and Behavioral Sciences, California Institute of Behavioral Neurosciences and Psychology, Fairfield, USA; 3 Dermatology, California Institute of Behavioral Neurosciences and Psychology, Fairfield, USA; 4 General Surgery, California Institute of Behavioral Neurosciences and Psychology, Fairfield, USA

**Keywords:** acetylcholine receptor antibody, anti dsdna antibody, anti-nuclear antibody, autoimmune neuromuscular disease, myasthenia gravis, neuromuscular disease, sle, thymectomy

## Abstract

Systemic lupus erythematosus (SLE) is a systemic autoimmune disease and myasthenia gravis (MG) is an organ-specific autoimmune disease, both may exhibit positive anti-nuclear antibodies and a female preponderance. They may have similar features and can coexist in a patient or precede one another.

This review article is based on electronic searches using PubMed as the primary database. Most of the articles used for this review were published in the last ten years with the exception of seven articles which were from 1995-2009. No guidelines have been followed. A total of 55 research articles were found related to the topic of this review article, and further scanning was done to eliminate some articles that did not meet the criteria.

The coexistence of autoimmune diseases has been reported in many cases. The prevalence of a second autoimmune disease is higher among patients with a primary diagnosis of autoimmune disease than the general population. The prevalence of SLE in MG patients or vice-versa is greater than the general population. The association has been hypothesized to many mechanisms: thymectomy resulting in loss of central tolerance and generation of autoantibodies, regulatory T cell dysfunction, the dysregulated function of Fas receptor (CD95), anti-malarial drugs directly affecting the neuromuscular junction, the role of chemokine CXCL13 and GM-CSF in the pathogenesis. The association is rare, and the presence of one should be closely followed for further progression into other diseases. More research work needs to be done for a clear conclusion.

## Introduction and background

Systemic lupus erythematosus (SLE) is an autoimmune disease of multisystem origin characterized by the presence of numerous auto-antibodies causing chronic inflammation. SLE is mostly seen (90% of cases) in women of childbearing age with a relapsing and remitting course, with severity varying from mild to rapidly progressing, accompanied by symptoms such as facial rash, fever, joint pain, photosensitivity, fatigue, and chest pain [1]. Myasthenia gravis (MG) is an organ-specific rare autoimmune disorder of neuromuscular junction where auto-antibodies are directed to the nicotinic acetylcholine receptor (nACHR), the muscle-specific tyrosine kinase (MuSK) or lipoprotein receptor-related protein 4 (LRP4) [2]. This blocks neuromuscular transmission resulting in muscle weakness which eventually improves with rest. It is commonly seen in younger females (less than 40 years) with features such as ocular symptoms (50-85% of patients), slurring of speech, facial weakness, difficulty in walking and lifting objects, and shortness of breath [3]. Autoimmune disorders affect approximately 5% of the population with female preponderance and a higher risk of being affected by a second autoimmune disease [4].

SLE and MG have similar features and precede one another or can coexist in a patient, which is a rare association [2]. They both have a higher incidence in the female population and both exhibit positive anti-nuclear antibodies [5]. The association between SLE and MG has been reported, which is seen in patients undergoing thymectomy for MG, for example, a case report of a 48-year-old female mentions the occurrence of SLE and secondary antiphospholipid syndrome (APS) 28 years post thymectomy for MG, with thymectomy being the precipitating factor [5]. Thymic abnormalities are commonly seen with MG patients; thus, thymectomy is considered as the first line of management in case of generalized or severe myasthenia as the thymus is known for autoantibody production [3, 6]. However, thymectomy does not have any effect in the case of established SLE cases [6].

According to a study in China, the prevalence of SLE-associated peripheral neuropathy (SLE-PN) was 1.5% in SLE patients (4924 total SLE patients) [7]. Around 10.1% of cases were diagnosed with myasthenia gravis among patients with SLE-PN [7]. The conclusion on the likely mechanism behind the association might be a useful tool in preventing the occurrence of the association by the application of proper screening methods or other preventive and treatment strategies. There are various proposed hypotheses for this association, but no precise mechanism is known. This review article is intended to understand the need for a conclusion which can help determine the outcome in the lives of the patients and thus help to take proper steps in improving the quality of life of the ones living with the diseases.

## Review

Methods

This is a traditional review article delineating the association between SLE and MG. The database used for this review article was PubMed. An article obtained from Medscape was also reviewed. Some related articles were excluded as full access was not provided.

The following keywords were used to view articles, case reports, and other research works, as summarized in Tables 1-2.

**Table 1:** Regular keywords SLE: Systemic lupus erythematosus

	Keyword	Database	Number of results
1.	Neuromuscular disease	PubMed	315217
2.	Anti dsDNA antibody	PubMed	34702
3.	SLE	PubMed	33297
4.	Myasthenia gravis	PubMed	18277
5.	Thymectomy	PubMed	9706
6.	Acetylcholine receptor antibody	PubMed	6965

**Table 2:** MeSH keywords

	Keyword	Database	Number of results
1.	Myasthenia gravis	PubMed	15323
2.	Anti-nuclear antibody	PubMed	14684
3.	Autoimmune neuromuscular disease	PubMed	9225

Results

The total number of results for various regular keywords were 31,5217 for neuromuscular diseases, 34,702 for anti dsDNA antibody, 33,297 for SLE, 18,277 for myasthenia gravis, 9,706 for thymectomy, and 6,965 for acetylcholine receptor antibody. Similarly, the total number of results for MeSH keywords were 15,323 for myasthenia gravis, 14,684 for anti-nuclear antibody, and 9,225 for autoimmune neuromuscular disease.

After a thorough screening, a total of 55 research articles were used to come to a conclusion that defined the co-existence between the two autoimmune diseases which is discussed in this review article, although some articles were excluded as it did not meet the criteria. There were no quality assessment tools used for this review article.

Discussion

Autoimmune diseases and neuropsychiatric manifestations

Autoimmune diseases are immune system-mediated disorders wherein the body, in response to a trigger, produces antibodies directed to the tissues and organs. The various etiology for the pathological process of auto-destruction are genetics, infections, hormonal, toxic chemicals, dietary, and idiopathic factors [8]. The prevalence of autoimmune diseases is approximately 5% of the population with a female preponderance [8]. The presence of autoantibodies may suggest possible disease development as it can be detected before the presentation of clinical features [9].

Neuropsychiatric (NP) symptoms are common manifestations seen in autoimmune disorders. Neuropsychiatric involvement in SLE (NPSLE) most frequently presents as a headache, but severe forms can occur that influence the disease outcome [10]. The American College of Rheumatology Research Committee in 1999 specified the 19 NP syndromes (12 CNS and 7 PNS) appearing in SLE; MG is one of the seven peripheral NPSLE [10]. It is estimated that while almost half of SLE patients are affected by NPSLE, the prevalence of MG as peripheral NPSLE is less than 1-2% [10]. Schwartz et al. also state the prevalence of MG as peripheral NPSLE as 0.2% [11].

The existing data gives us an idea about the high prevalence of neuropsychiatric manifestations in SLE and other autoimmune diseases. However, the prevalence of MG is less than 1-2%. The subsequent associated disease (MG in this case) may present with overlapping symptoms masking its detection; hence any indicator should be promptly evaluated.

Myasthenia gravis and associated autoimmune diseases

MG is an organ-specific autoimmune disease with the presence of specific antibodies directed to the nicotinic acetylcholine receptor (nACHR), the muscle-specific tyrosine kinase (MuSK) or lipoprotein receptor-related protein 4 (LRP4) [2]. The specific antibodies are directed to the postsynaptic membrane receptors, resulting in the disturbance of neuromuscular transmission with features such as fatigability and weakness of various muscle groups during the activity which improves with rest [8]. Ocular symptoms comprise approximately 15% of the patients with MG [12].

Based on the 55 studies conducted within 1950-2007, the incidence and prevalence rates were calculated to be 5.3 per million person-years and 77.7 cases per million, respectively [13]. The same study also provides a mortality rate of 0.06-0.89 per million person-years [13]. Another cohort study conducted, 1996-2013, showed an incidence rate of 2.7-2.8 per 100,000 population and a prevalence rate of 16.6 (in 1996) increased to 32.0 (in 2013) per 100,000 population [14].

There is an increased incidence of associated autoimmune diseases in patients having MG such as rheumatoid arthritis, SLE, Hashimoto thyroiditis, Graves' disease, and pernicious anemia, the frequency ranging in between 8.7-25% [8, 15-18]. The most common autoimmune disease associated with MG is thyroid disease with a prevalence of 5-10%, while only 0.2% of patients with autoimmune thyroid are diagnosed with MG [15]. Thyroiditis, SLE and rheumatoid arthritis are said to be the most common associated conditions, thyroiditis being most prevalent [12]. There is a sex ratio of 1.9:1 (F>M) and a mean age of 51.0 years in a study of 127 patients with an established diagnosis of MG; 15% had another autoimmune disease, and 0.8% with SLE [8].

MG is a rare condition, but the prevalence of the disease has been increasing over the years, affecting bimodal age groups and women more frequently than men. The increasing trend in the occurrence as compared to the past may be due to advanced investigation procedures along with early recognition and diagnosis.

SLE and its association with MG

SLE involves multiple systems with various antibodies that contribute to the inflammatory state of the body, predominantly affecting young women [2]. Unlike MG, SLE is a systemic disorder affecting almost any organ system (deposition of immune complexes) and diagnosed by laboratory findings or clinically by having at least 4/11 of the American College of Rheumatology (ACR) listed criteria [1]. Women, especially of childbearing age, comprise the majority of SLE cases (90%) with a variable severity scale [1]. The prevalence rate of SLE has a wide range, varying from 37 to 178 cases per 100000 [19].

SLE coexistent with MG is rare and thought to be coincidental; however, reported cases do exist. The similarities shared by these diseases are higher prevalence among young women, positive anti-nuclear antibodies, and relapsing and remitting course [20-21]. There have been reports showing a majority of cases (61.5%) where MG preceded SLE but cases where SLE precede MG have also been reported [22-23].

A group of 132 MG patients was evaluated and found the prevalence of SLE to account for 3.78%, five having SLE (4/5 females), and 11 ANA positive without any SLE symptoms [23]. Sthoeger et al. reported a higher prevalence of 7.7% of SLE in patients with MG in a study of 78 patients [24]. Another study observed a group of 380 SLE patients and the prevalence of MG was stated as 0.25% in that population group, whereas the prevalence in the general population is 0.02% [25-26]. Jallouli et al. conducted a cohort study of 1300 SLE patients, 17 (1.3% and all women) of which were diagnosed with both MG and SLE [22]. Their research showed a higher prevalence of MG in patients with SLE patients (1.3%) and the occurrence of SLE in MG patients (8%) which was comparatively greater than the general population [22].

A case series reported four patients for SLE-MG overlap syndrome; two had thymectomy done after the establishment of the diagnosis MG and pyridostigmine continued, whereas the third case didn't respond well to pyridostigmine upon initial diagnosis of MG and was later diagnosed with SLE-myositis overlap syndrome [6]. This series demonstrates the different treatment strategies for different cases. The following table displays the events and the treatment for the reported case series by Minchenberg et al. (Table 3) [6].

**Table 3:** Details of the case series M: male; F: female; MG: myasthenia gravis; SLE: systemic lupus erythematosus; APS: antiphospholipid syndrome

Variables/Events	Case 1	Case 2	Case 3	Case 4
Age/sex	62/F	56/F	57/M	58/F
MG	Present, 29 years prior	Present, 46 years prior	Present, three years prior	Absent (Initially diagnosed as SLE)
Treatment (initial)	Thymectomy, pyridostigmine	Thymectomy, pyridostigmine	Cholinesterase inhibitor failed	Hydroxychloroquine (self-withdrawal after one year)
SLE	Present	Present	Present; APS- Present	MG - Present; APS - Present
Treatment (later)	Hydroxychloroquine	Hydroxychloroquine	Mycophenolate mofetil and hydroxychloroquine	____________

These findings are important to keep in mind with respect to the possible differential diagnoses such as SLE, APS, and myositis when patients with MG do not respond to cholinesterase inhibitors [6]. When comparing one autoimmune disease preceding the other, it can be seen that the occurrence of SLE following MG is up to 8%, in contrast to MG following SLE being 1.3% [22]. One of the reasons for this dissimilarity can be the overlapping symptoms seen in two and MG is also one of the peripheral NPSLE.

Immunological association in the pathogenesis 

An alpha chemokine has been noted in the pathogenesis of both diseases, as they are involved in the activation of various cells (monocytes, dendritic cells, T, B, NK cells, eosinophils, and basophils) and also partake in angiogenesis [20]. CXCL13 is a chemokine that activates B and T lymphocytes, which further contributes to the pathogenesis of SLE and MG, respectively [23]. A higher level of serum and renal concentrations of CXCL13 were found in patients with SLE than a control group [27]. Similar conclusions have been drawn between MG and CXCL13, where higher levels of CXCL13 were detected in patients with MG, in the thymus (overexpression) and serum of patients not treated with corticosteroids [28-29]. Therefore, raised levels of CXCL13 can contribute to the development of both autoimmune diseases.

Granulocyte-macrophage colony-stimulating factor (GM-CSF), found endogenously as well as exogenously, is a common factor between the two autoimmune diseases. It can be produced endogenously by various cell types, for example, T cells, B cells, monocytes, macrophages, mast cells, fibroblasts, vascular endothelial cells [30]. At the same time, exogenous GM-CSF is used as a treatment to help synthesize bone marrow-derived macrophages and granulocytes [30]. In a study by Sheng et al., the administration of GM-CSF reduced the number of circulating antibodies against the AchR and proliferation of T cells [31]. The same study also shows GM-CSF to reduce the apoptosis of neutrophils improving their overall function, which imposes its administration as a treatment option in patients with SLE [31]. According to this study (an experimental study done on mice), GM-CSF has some protective functions in the line of prevention as well as treatment. This has yet to be supported by additional research works.

Thymectomy and increasing risk for autoimmune diseases

The thymus is the site for T cell maturation, pathological processes in thymus cause cell dysfunction and activation of auto-reactive CD4+ T lymphocytes which leads to autoantibody production after interaction with B lymphocytes [20]. Regulatory T lymphocytes are responsible for halting the autoimmune process by inhibiting the activity of CD4+ T lymphocytes. Regulatory CD4+ CD25+ T lymphocyte deficiency or dysfunction has been considered a factor for the development of connective tissue diseases and thus the development of MG and SLE [20].

There have been cases reported stating the occurrence of systemic autoimmune disorders in patients who underwent thymectomy as the treatment for MG. Thymectomy is another therapeutic option in MG as the thymus is considered the source of autoantibody production [6]. Thymectomy results in loss of central tolerance to its antigen and excessive generation of autoantibodies [22].

The common symptom presentation in the case of post thymectomy SLE cases was polyarthritis and polyarthralgia with laboratory findings showing mild T-cell lymphopenia, hypergammaglobulinemia, and B cell hyper-reactivity [21]. Thymectomy can serve to bring out many autoimmune diseases such as SLE, Hashimoto’s disease, antiphospholipid syndrome, idiopathic portal hypertension, cutaneous vessel vasculitis [21]. The following table shows the concomitance and prevalence of SLE and MG as seen in various articles (Table 4) [8, 22-24].

**Table 4:** Concomitance and prevalence of SLE and MG n: total number; Av: average; M: male; F: female; MG: myasthenia gravis; SLE: systemic lupus erythematosus

SN	MG (n)	SLE (concomitant)	Gender ( M: F)	Age at diagnosis (Av)	Thymectomy associated with SLE	Prevalence	Reference
1	127	1	0:01	48.8	-----------------	0.80%	[8]
2	132	5	1:04	24-58	2/5	3.78%	[23]
3	78	6	0:06	44.5	2/6	7.70%	[24]
SN	SLE (n)	MG (concomitant)	Gender ( M: F)	Age at diagnosis (Av)	-----------------	Prevalence	Reference
1	1300	17	0:17	34.5	-----------------	1.30%	[22]

Regulatory T cell dysfunction in the thymus, dysregulated expression of FAS (CD95) and anti-malarial drug usage resulting in symptoms such as muscle weakness are some other speculated mechanisms defying the association between the two autoimmune diseases [2].

Hydroxychloroquine was assumed to initiate MG, but patients treated with hydroxychloroquine for SLE had milder forms of MG as compared to others [22-23]. Anti-malarial drugs directly affect the neuromuscular junction, commonly resulting in neuromyopathy, with muscle biopsies showing atrophic muscle fibers [22]. Hydroxychloroquine treatment can result in ocular symptoms and symmetrical muscle weakness, hence when symptoms persist despite treatment withdrawal, MG should be ruled out [2].

Limitations

This review article has been derived from a paucity of research papers, case reports, and review articles. The variable prevalence rates (0.8-7.7%) among different studies may be due to limited sample size, failure of early detection, lack of proper research techniques, or procedural issues. The majority of the articles are either retrospective studies or case reports, and therefore cannot be used to track the progression of the disease. The pathophysiology behind the association is theorized to various mechanisms, and interlinks such as thymectomy increasing the risk for the occurrence of SLE, presence of chemokines leading to the pathogenesis, and genetic factors but do not suffice a conclusion. It may be because of the limited number of studies available to analyze. In addition, the prognosis of the diseases when parallel has not been mentioned, yet it is a crucial aspect to look in. This could perhaps contribute to disease management. 

## Conclusions

The sole purpose of this review article is to provide readers and upcoming researchers the information on the association between MG and SLE. Some reviews have stated the concomitance to be coincidental while some state the possibility of relying on various mechanisms with no conclusive data. The reviewed literature shows a higher prevalence of SLE in MG patients and vice versa in comparison to the general population. A greater prevalence of SLE is also seen in patients who have undergone thymectomy (variable number of years after the surgery). The associated autoimmune disorder not being initially detected and mistaken for the course of the existing disease may be the reason for late detection. This may result in unusual outcomes, relapse cases, treatment failures, and an increase in the severity of the condition.

CXCL13, a chemokine, has also been speculated to have a role in the development as higher levels were found in patients with SLE and MG. Similarly, the role of GM-CSF is thought to be protective, and deficiency of GM-CSF may be linked to the mutual pathogenesis of the two autoimmune diseases. Further studies are warranted to clear concepts about the precise pathogenesis. The coexistence of MG and SLE is a complex one and may result in greater severity of the disease progression. A close follow-up is mandatory in preventing possible outcomes and treatment accordingly, especially in the high-risk population (women), and post-thymectomy cases. More research work is required on a larger scale to clarify these theories in hopes to yield a better management strategy.
